# Targeting HER2-breast tumors with scFv-decorated bimodal nanoprobes

**DOI:** 10.1186/s12951-018-0341-6

**Published:** 2018-02-21

**Authors:** Christophe Alric, Katel Hervé-Aubert, Nicolas Aubrey, Souad Melouk, Laurie Lajoie, William Même, Sandra Même, Yann Courbebaisse, Anastasia A. Ignatova, Alexey V. Feofanov, Igor Chourpa, Emilie Allard-Vannier

**Affiliations:** 1 EA6295 ‘Nanomédicaments et Nanosondes’, Université de Tours, 37200 Tours, France; 2 ISP, Université de Tours, INRA, UMR 1282, Equipe BIOMédicaments Anti-Parasitaires, 37380 Nouzilly, France; 3 GICC ‘Groupe Innovation et Ciblage Cellulaire’, Université de Tours, Equipe FRAME - Fc Récepteurs, Anticorps et MicroEnvironnement, 37032 Tours, France; 4 CBM, CNRS, UPR4301, Equipe Complexes Métalliques et IRM pour applications biomédicales, 45071 Orléans, France; 5 Bertin Pharma Orléans, 45071 Orléans, France; 6 Shemyakin-Ovchinnikov Institute of Bioorganic Chemistry, Russian Academy of Sciences, ul. Miklukho-Maklaya, 16/10, Moscow, 117997 Russia; 7 Biological Faculty, Lomonosov Moscow State University, Vorobyevi Gori 1, Moscow, 119992 Russia

**Keywords:** Breast tumor, Human Epithelial growth Receptor 2 (HER2), Iron oxide nanoparticle, Cyanine 5.5, Single chain variable fragment (scFv), Magnetic Resonance Imaging (MRI)

## Abstract

**Background:**

Recent advances in nanomedicine have shown the great interest of active targeting associated to nanoparticles. Single chain variable fragments (scFv) of disease-specific antibodies are very promising targeting entities because they are small, not immunogenic and able to bind their specific antigens. The present paper is devoted to biological properties in vitro and in vivo of fluorescent and pegylated iron oxide nanoparticles (SPIONs-Cy-PEG-scFv) functionalized with scFv targeting Human Epithelial growth Receptor 2 (HER2).

**Results:**

Thanks to a site-selective scFv conjugation, the resultant nanoprobes demonstrated high affinity and specific binding to HER2 breast cancer cells. The cellular uptake of SPIONs-Cy-PEG-scFv was threefold higher than that for untargeted PEGylated iron oxide nanoparticles (SPIONs-Cy-PEG) and is correlated to the expression of HER2 on cells. In vivo, the decrease of MR signals in HER2+ xenograft tumor is about 30% at 24 h after the injection.

**Conclusions:**

These results all indicate that SPIONs-Cy-PEG-scFv are relevant tumor-targeting magnetic resonance imaging agents, suitable for diagnosis of HER2 overexpressing breast tumor.

**Electronic supplementary material:**

The online version of this article (10.1186/s12951-018-0341-6) contains supplementary material, which is available to authorized users.

## Background

Despite many advances in the treatment of cancer, developing novel approaches for the accurate detection of cancer and for targeted therapies based on cancer-specific markers is still in the news. Nanomedicines could be ideal candidates to achieve this goal due to their unique properties compared to traditional drug formulations or imaging agents. However, finding a relevant strategy to target selective tumors by using nanomedicine has been a big challenge so far [1]. In this context, various nanoparticles (NPs) are developed for targeted delivery of diagnostic/therapeutic agents to the tumor sites, intended to result in greater efficacy and less side effects. Two strategies are still studied intensively, passive and active targeting [2]. Passive targeting is based on the diffusion of the NPs into the tumor by the so-called enhanced permeability and retention (EPR) effect [3, 4]. Unfortunately, this approach alone is not sufficient since it suffers from several limitations. The most important limitation is that targeting cancer cells using the EPR effect is not feasible in all tumors because the degree of tumor vascularization and porosity of tumor vessels can vary with the tumor type and status [5, 6]. Active targeting utilizes biological ligands attached to the NPs to recognize overexpressed biomarkers on tumors. In this strategy, two targets can be distinguished: (i) cancer malignant cells and (ii) tumor microenvironment [7, 8]. Attachment of cell-targeting ligands onto NPs surface has provided further advantages such as increased cellular uptake, reduced side effects and better therapeutic efficacy in vitro as well as in vivo [8–10].

Numerous targeting moieties are available for NPs functionalization, including small molecules, sugars, fatty acids, peptides, proteins, aptamers and monoclonal antibodies (mAbs) [11, 12]. Not only the choice of the correct targeting ligand is crucial but also the conjugation chemistry used to attach it is essential and can impact the therapeutic outcome of such targeted nanodevices [11]. Within the targeting ligands extensively studied, there are antibody moieties, and especially engineered antibody fragments [13] such as single chain variable fragment (scFv) [10], disulfide-stabilized Fv antibody fragment (ds-Fv), ds-scFv, single chain antibodies (sdAb) and diabodies. These antibody fragments retain at least one antigen-binding region and are characterized by their simple structure (lack of an Fc domain) and their molecular weight (25–50 kDa) compared to whole mAbs (150 kDa). When applied to nanoparticle functionalization, these two properties lead to higher loading capacity and better orientation of the antibody fragment and lower immunogenicity.

Our group recently designed a new generation of cancer-targeting magnetic nanoprobes based on SuperParamagnetic Iron Oxide Nanoparticles (SPIONs) coated with polyethylene glycol (PEG). SPIONs are well known as MRI contrast agents useful for cancer imaging [14, 15]. On this PEG layer, SPIONs were functionalized with a specifically designed scFv directed against human epidermal growth receptor 2 (HER2) [16]. The membrane protein HER2 (also known as ErbB-2, Neu, CD340) is closely associated with malignancy, and is highly expressed in various tumors including mammary tumors [17, 18]. Our targeted nanosystem presents the following advantages compared to the others: (i) a site-selective maleimide-thiol coupling to achieve optimal orientation of the scFv targeting moieties on the surface of PEGylated SPIONs; (ii) a moderate number of 7 antibody fragments per NP to preserve both neutral surface and small size compatible with their immune stealthiness, (iii) an IRM/optic bimodality as our nanoprobes are labelled with a deep-red fluorescent dye (cyanine 5.5 hereafter called Cy) covalently bound to the iron oxide core. The advantage of using the deep-red absorbing and near-infrared emitting Cy was related to the fact that both light absorption and emission by cells and tissues in this spectral region is very low.

In this present paper, we investigated the potential of these targeted imaging agents for the accurate detection of HER2 breast cancer in the context of diagnosis (evaluation of HER2 status) or support for surgery. After a rigorous characterization of our HER2-positive breast cancer model, we especially investigated the HER2 protein binding affinity of our nanoprobes SPION-Cy-PEG-scFv, their cellular uptake and in vivo tumor targeting. Thanks to their bimodal properties, we alternately employed methods to track either the magnetic core of the nanoprobes (IRM, TEM) or its organic fluorescently labelled shell (FACs, confocal imaging).

## Conclusion

In conclusion, we developed SPIONs-Cy-PEG-scFv nanosystems that show high affinity to HER2 overexpressed on breast cancer cells in vitro. Their fluorescent labelling made possible to follow, both qualitatively and quantitatively, their uptake in the cancer cells. In vivo, the SPION-Cy-PEG-scFv allowed a selective MRI labelling of HER2-positive tumors. The presented results indicate that these nanoparticles functionalized with antibody fragment have a promising potential as targeted imaging agent for use in diagnostics of malignant tumors. Furthermore, it should be possible to exploit them also in anticancer therapy, as drug carriers and/or hyperthermia substrates. The theranostic properties of these nanosystems will be in a focus of our next studies.

## Results and discussion

### Nanoprobe physico-chemical characteristics and biofunctionality

**Fig. 1:**
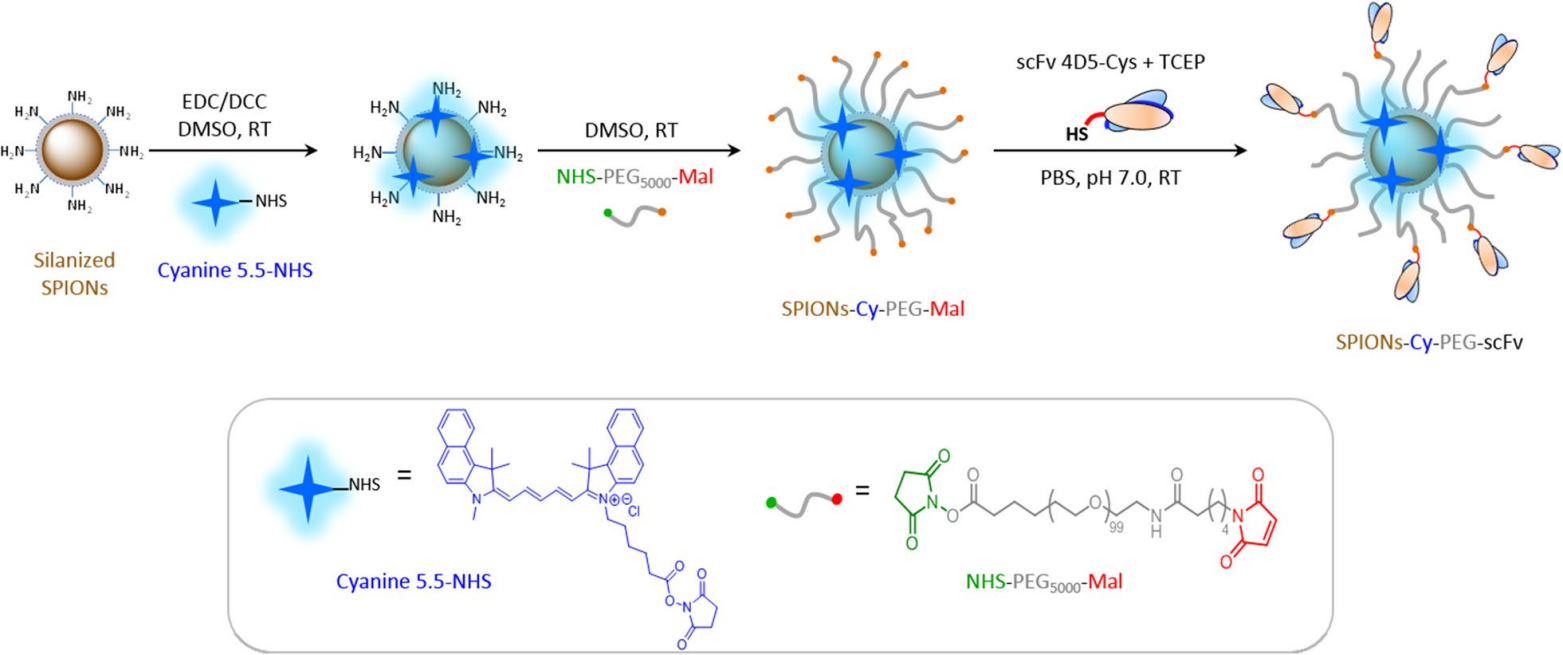
Schematic representation of SPIONs-Cy-PEG-scFv synthesis

The preparation of fluorescent PEGylated SPIONs functionalized with scFv anti-HER2 (SPION-Cy-PEG-scFv) includes three steps, as shown in Fig. 1. The synthesis starts with silanized SPIONs that were obtained by reaction between silane molecules and the surface hydroxyls groups of SPIONs [19]. The first step consists to introduce the fluorophore Cyanine 5,5-NHS (Cy) at the nanoparticle surface. To not affect the colloidal stability of nanoparticles and to protect the fluorophore from further quenching by external interactions, Cy was directly grafted on the SPION core and thus buried within a PEG polymeric layer. The NHS group of Cy reacts with primary amine groups at the surface of silanized SPIONs. The excess of primary amines then reacts with NHS-PEG_5000_-maleimide upon the second step of the process. The excess of cyanine dye and NHS-PEG-mal were eliminated by dialysis for 48 h at 4 °C in order to preserve the maleimide functions. In the third step, the scFv fragment 4D5 was grafted on the surface of SPIONs-Cy-PEG according to our protocol previously described [16]. The oriented coupling of scFv at the surface of PEGylated SPIONs was performed via the formation of stable covalent thioether bonds between the terminal cysteine residue introduced on the hexahistidine tag of the antibody fragment and maleimide terminal groups of the polymer chains. The SPIONs-Cy-PEG-scFv were purified from free scFv by dialysis and were concentrated using centrifugal concentrator. The efficiency of purification process was controlled by ELISA (Additional file 1: Fig. S1). Results show that none scFv was detected in filtrate confirming the absence of free scFv and consequently the efficiency of dialysis.

The physico-chemical properties of SPIONs-Cy-PEG and SPIONs-Cy-PEG-scFv were compared. The hydrodynamic diameter in PBS at pH 7.4 were 65.8 ± 2.2 nm and 70.5 ± 1.8 nm respectively with a polydispersity index below 0.2 (Additional file 1: Fig. S2A). The zeta potential were from − 4.6 to − 5.0 mV at physiological pH. So, the presence of scFv at the surface of nanoparticles did not perturb the physicochemical properties of nanoparticles, no significant increase of the D_H_ was observed and the surface of SPIONs remained almost neutral. The quantity of scFv covalently bound to SPIONs-Cy-PEG-scFv was estimated to be around 7 scFv per SPION [16]. Others groups conclude that the functionalization of SPIONs with scFv do not significantly increase their diameter as scFv are relatively small entities of approximately 5 nm [20]. As noted previously [21], the presence of Cy dye was confirmed by the strong fluorescence signal recorded from the functionalized SPIONs suspended in water (Additional file 1: Fig. S2B). The shape of the Cy spectra obtained for nanoparticles with or without scFv is strictly the same which indicates identical polarity of the fluorochrome molecular environment (presumably that of hydrated PEG) in both batches. This result is in favor with the assumption that the chromophore should be buried under the protective polymeric layer. In contrast to the NPs where the fluorophores are attached to the external polymeric surface, our NPs labelling strategy offers several advantages because it allows to avoid and/or to reduce the following undesired phenomena that may occur upon the NP tracking in biological media: (i) degradation/detachment of the fluorophore from the NP by cleavage; (ii) emission quenching by interactions/environment changes; and (iii) loss of targeting/stealthiness properties because of interference of the fluorophore molecules.

**Fig. 2:**
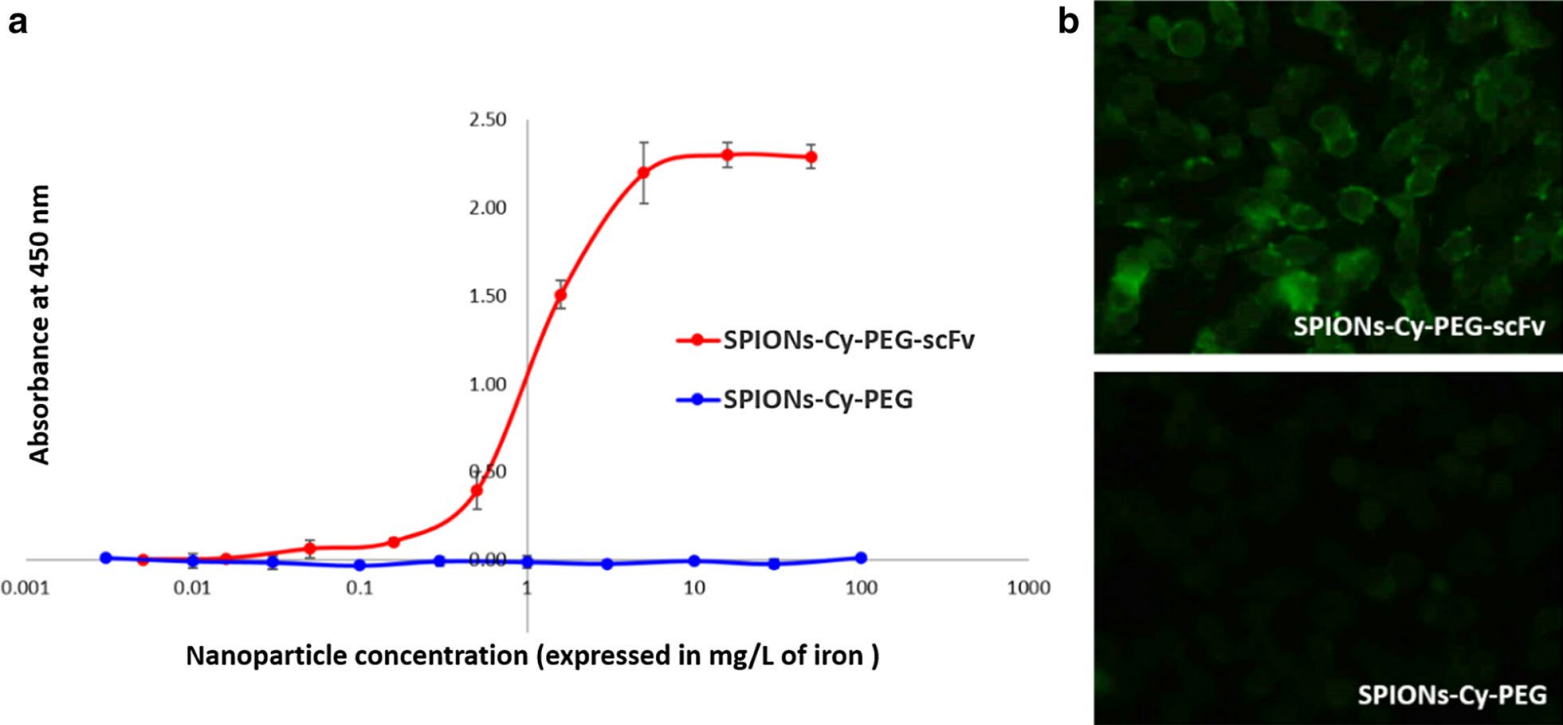
Functionality of SPIONs-Cy-PEG-scFv regarding HER2 proteins. **a** Indirect ELISA test of the immunoreactivity of SPIONs-Cy-PEG-scFv (red curve) vs. SPIONs-Cy-PEG (blue curve). **b** Immunofluorescence images of SK-BR3 breast cancer cells incubated in the presence of SPIONs-Cy-PEG-scFv and SPIONs-Cy-PEG (detection with PpL-FITC)

Lastly, the ELISA assay performed on increasing concentrations of SPIONs-Cy-PEG and SPIONs-Cy-PEG-scFv confirmed the functionality of the scFv grafted. The scFv revelation was made by Protein L (PpL), which is able to bind to some kappa light chain variable domains without interfering with the antigen-binding site. This characteristic has the great advantage to detect antibody fragments such as scFv [22], which are devoid of constant domains. The absorbance of the substrate of protein-l-HRP increased with the concentration of SPIONs-Cy-PEG-scFv whereas the absorbance remained negligible for SPIONs-Cy-PEG (Fig. 2a). Thus, the covalent conjugation of the scFv at the surface of the nanoprobes did not hinder its antigen binding ability. Kanazaki et al. showed that scFv anti-HER2 associated to iron oxide NPs (IONP) exhibited higher affinity (measured by surface plasmon resonance—SPR) compared to trastuzumab and to peptide-IONPs, whereas the Kd of each targeting moiety alone is better for trastuzumab [23]. This suggested that orientation of anti-HER2 moiety is an important factor for the binding affinity to iron oxide NPs.

The immunoreactivity of the SPIONs-Cy-PEG-scFv was additionally confirmed by immunofluorescence labelling of HER2-overexpressing breast cancer cells SK-BR3. As we can see on Fig. 2b, plasma membranes of the SK-BR3 overexpressing cell lines incubated with SPIONs-Cy-PEG-scFv appear fluorescent whereas it remain dark with SPIONs-Cy-PEG. That confirms a specific recognition of the scFv-nanoprobe to HER2 receptors. This suggests that the scFv molecules attached on the NPs surface were not denatured and were favorably exposed to the exterior thanks to the site-selective coupling [16].

### HER2 quantification and cellular uptake

**Fig. 3:**
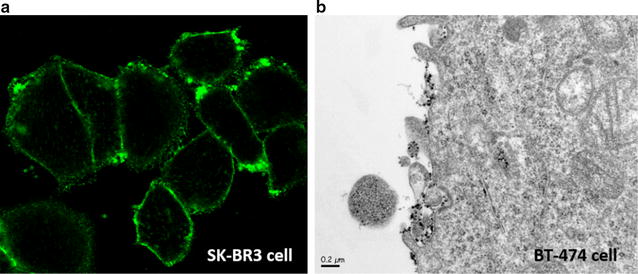
Qualitative evaluation of HER2 proteins on breast cancer cell lines. **a** SPE-CLSM data on SK-BR3 cells after the incubation of anti-human CD340-Alexa Fluor^®^ 488. **b** Electronic microscopies data on BT-474 cell incubated with anti-ErbB2 MicroBeads (Miltenyi Biotec)

What about the targeted HER2 receptor? It has been demonstrated previously in several papers that SK-BR3 and BT-474 human breast cancer cells express high levels of HER2 receptors [24, 25]. Thus, HER2 receptors location on cells was investigated on SK-BR3 and BT-474 cells by confocal microscopy respectively and Transmission Electron Microscopy (TEM). Figure 3a shows that plasma membranes of SK-BR3 cells were illuminated further to the incubation of fluorescent anti-HER2 antibodies. By the same, dark zones were clearly identified at the cell periphery further the incubation of anti-HER2 antibodies coupled with nano-sized MACS^®^ Microbeads (Miltenyi Biotec) (Fig. 3b). MACS MicroBeads are 50-nm magnetic particles conjugated to specific antibodies, designed for magnetic cell sorting. Here, we used these nanoforms to detect HER2 location on TEM. These two microscopy modalities confirm that HER2 receptors were mainly localized at plasma membranes of some breast cancer cells and that scFv anti-HER2 should interact with them.

**Fig. 4:**
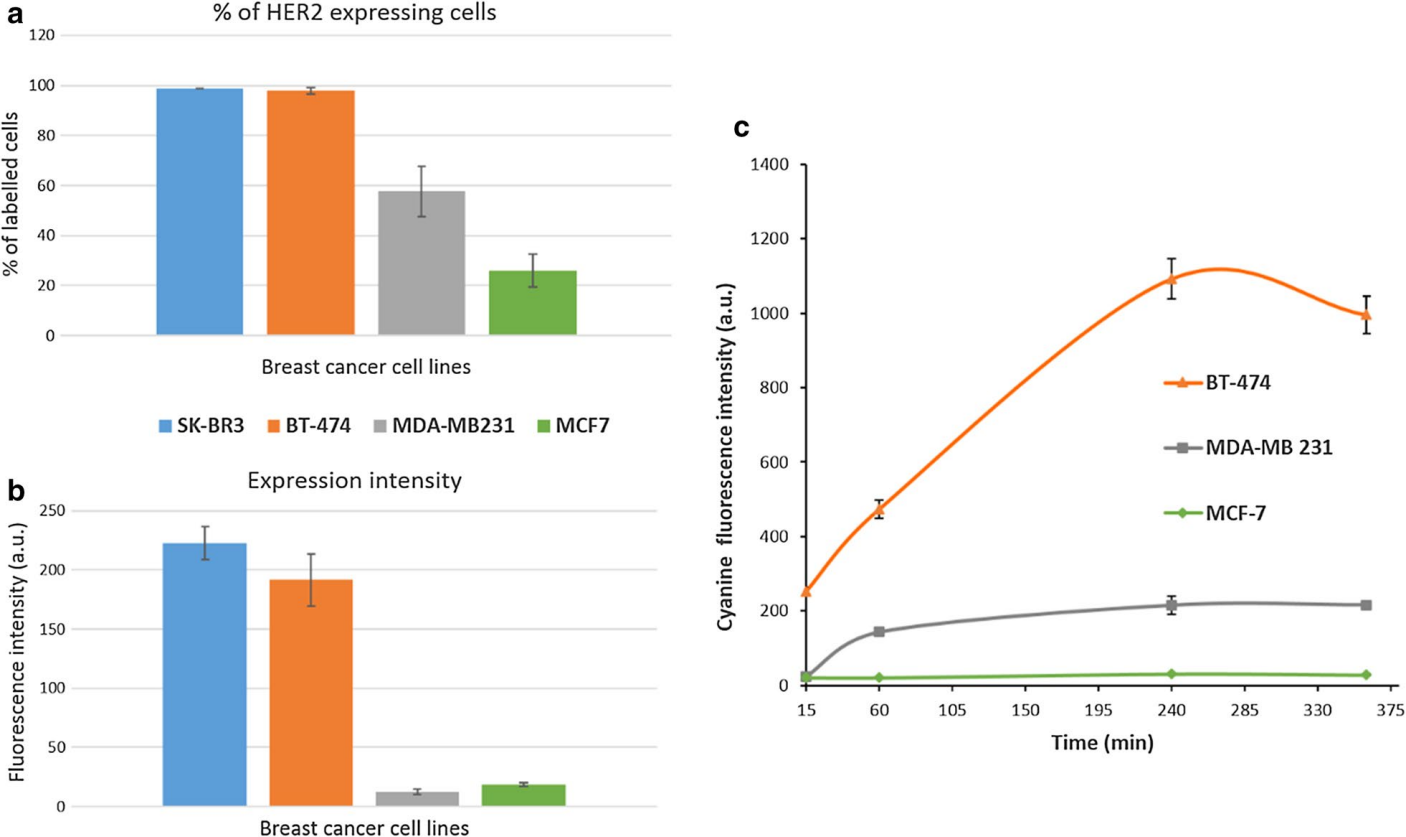
Quantitative evaluation of HER2 proteins on breast cancer cell lines. Flow cytometry data giving the percentage of positive cells (**a**) and the mean fluorescence intensity (MFI) (**b**) on SK-BR3, BT-474, MDA-MB231 and MCF-7 breast cancer cells. (**c**) Uptake of scFv-nanoprobes by BT-474, MDA-MB231 and MCF-7 cells according time from 15 to 360 min

When we talk about active targeting, numerous study demonstrated that the uptake of targeted NPs is enhanced when the cells overexpress the accurate receptor [26]. However, investigations of possible correlation to the level of the receptor expression are rare, although are critically important. Thus, prior to testing nanosystems on cells, we quantified the HER2 expression in four human breast cancer cell lines; BT-474, SK-BR3, MDA-MB231 and MCF-7 by flow cytometry. Two parameters were evaluated: the percentage of HER2 positive cells that represent the presence of HER receptors at the cell surface and the mean fluorescence intensity (MFI) showing the level of expression of this receptor. We found that 99 and 98% of SK-BR3 and BT-474 cells respectively have a very high level of the HER-2 receptor expression (Fig. 4a). Indeed, the MFI was about 222 ± 14 u.a. for SK-BR3 cells and about 191 ± 22 u.a. for BT-474 cell lines (Fig. 4b). In contrast, only 26% of MCF-7 cells expressed the receptor with a MFI around 19 ± 2 u.a. Moreover, 58% of MDA-MB231 cell line expressed the HER2 receptor but with a very low level of fluorescence as MFI was around 13 ± 2 u.a. To sum up, SK-BR3 and BT-474 were considered as HER2 overexpressing cell lines (HER2++), MDA-MB231 and MCF-7 were called HER2 negative (HER2−) even though their level of expression was not totally zero, especially for MDA-MB231. Taking into account this quantification, SPIONs-Cy-PEG-scFv were then incubated on BT-474, MDA-MB231 and MCF-7 cells for 10–360 min. The mean fluorescence intensity (MFI) steadily increased with time for all cell lines and started saturating after 240 min (Fig. 4c). For MCF-7 (HER2−), the amount of SPIONs-Cy-PEG-scFv able to penetrate the cells is very few and the increase according time is very low. For BT-474 cells (HER2++), at very early times up to 15 min, a large quantity of NPs were internalized by the cells. This amount increased until 240 min and stagnate until 360 min that traduce a saturation phenomenon. For MDA-MB231, the uptake of SPIONs-Cy-PEG-scFv during the first hour is quite moderate and start saturating after 100 min. This experiment means that the uptake of SPIONs-Cy-PEG-scFv by breast cancer cells is proportional to their HER2 level of expression. These results confirm the interest of nanoprobe functionalization with scFv anti-HER2 in the aim of HER2 overexpressing breast cancer imaging or therapy.

### In vitro targeting on BT-474 breast cancer cells

**Fig. 5:**
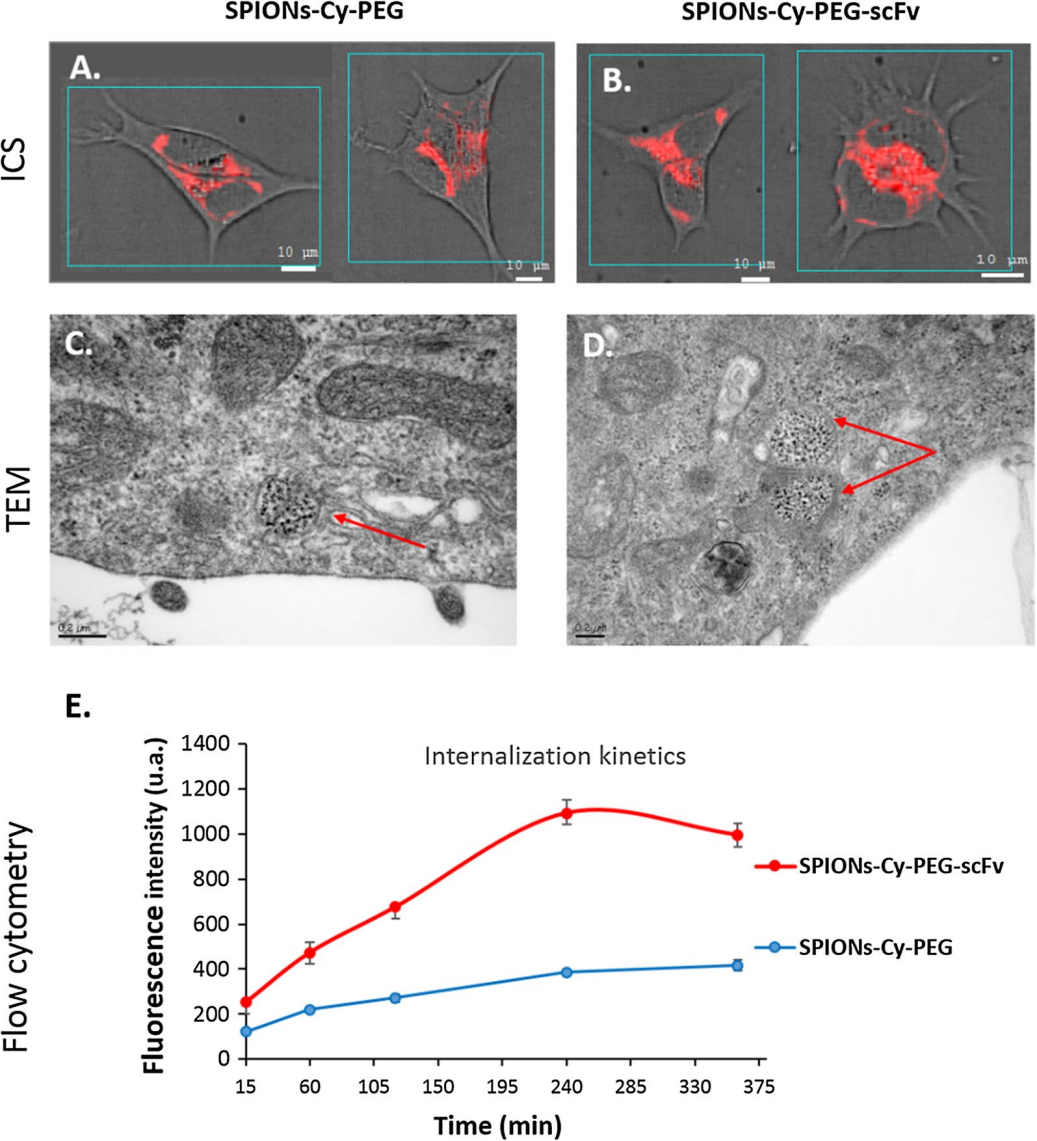
Endocytic pathway of SPIONs-Cy-PEG and SPIONs-Cy-PEG-scFv in HER2++ BT-474 breast cancer cells. Confocal spectral imaging data on fluorescence of the Cy-labeled nanoprobes, SPIONs-Cy-PEG (**A**) and SPIONs-Cy-PEG-scFv (**B**) in BT-474 cells (1 h–37 °C–150 mg/L of iron), **C**, **D** electronic microscopies data on SPIONs-Cy-PEG and SPIONs-Cy-PEG-scFv uptake by BT-474 cells (4 h–37 °C–180 mg/L of iron), **E** flow cytometry data on BT-474 cells after the incubation of Cy-labelled nanoprobes with or without anti-HER2 scFv (15–360 min–37 °C–150 mg/L of iron in PBS)

We wanted to study also the specificity of the targeting with scFv by comparing the cellular trafficking of SPIONs-Cy-PEG and SPIONs-Cy-PEG-scFv. According to the CSI data, which exploits the full fluorescence spectrum from numerous points of an optical section of the cell, both nanoprobes were taken up into the cytosol of BT-474 cells after 1 h of incubation (see the maps shown in red, Fig. 5A, B). The fluorescent nanoprobes accumulated in perinuclear cytosolic locations, which are logically assignable to endo-lysosomal compartments. That means that scFv capped nanoprobes penetrate into the cells by endocytosis which is expected for NPs of about 70 nm in size [27]. The endocytic pathways were confirmed using TEM analysis on BT-474 cells pre-incubated with SPIONs-Cy-PEG and SPIONs-Cy-PEG-scFv for 4 h (Fig. 5C, D). On TEM images, nanoprobes were found in endocytic vesicles surrounded by membranes, which is consistent with endosome structures. CSI and TEM give us qualitative data that were not able to make the difference between SPIONs-Cy-PEG and SPIONs-Cy-PEG-scFv. To go further, cytometry data performed on BT-474 cells give us quantitative information about the amount of nanoprobes penetrating the cells in function of time. SPIONs-Cy-PEG are able to penetrate BT-474 cells, even though the absence of targeting ligand, which is not surprising taking into account their small size. Moreover, Fig. 5E shows internalization kinetics of SPIONs-Cy-PEG versus SPIONs-Cy-PEG-scFv on BT-474 HER2++ breast cancer cells. Even with very short incubation times (60 min), SPIONs-Cy-PEG-scFv penetrate the cells more than twice compared to SPIONs-Cy-PEG. After 240 min, the uptake of SPIONs-Cy-PEG-scFv was threefold higher than that for SPIONs-Cy-PEG.

### In vivo targeting of HER2 ectopic breast tumor

To confirm these promising in vitro results, three groups of BalB-C female nude mice were injected subcutaneously with 10 million of BT-474 cells to develop HER2 ectopic breast tumors. Six weeks later, the expression of HER2 proteins was checked on ectopic tumors to be sure that the expression of the receptor is maintained in vivo. The quantification of HER2 was performed by flow cytometry and compared to BT-474 only grown in vitro. Results show that the expression of HER2 is maintained on BT-474 cells implanted on mice flank for 6 weeks (Additional file 1: Fig. S3). The percentage of HER2 positive cells and the MFI for cells grown in vivo were unchanged compared to BT-474 grown in vitro. No significant difference can be observed between the two conditions showing that our ectopic xenograft is a relevant in vivo model for HER2 targeting.

**Fig. 6:**
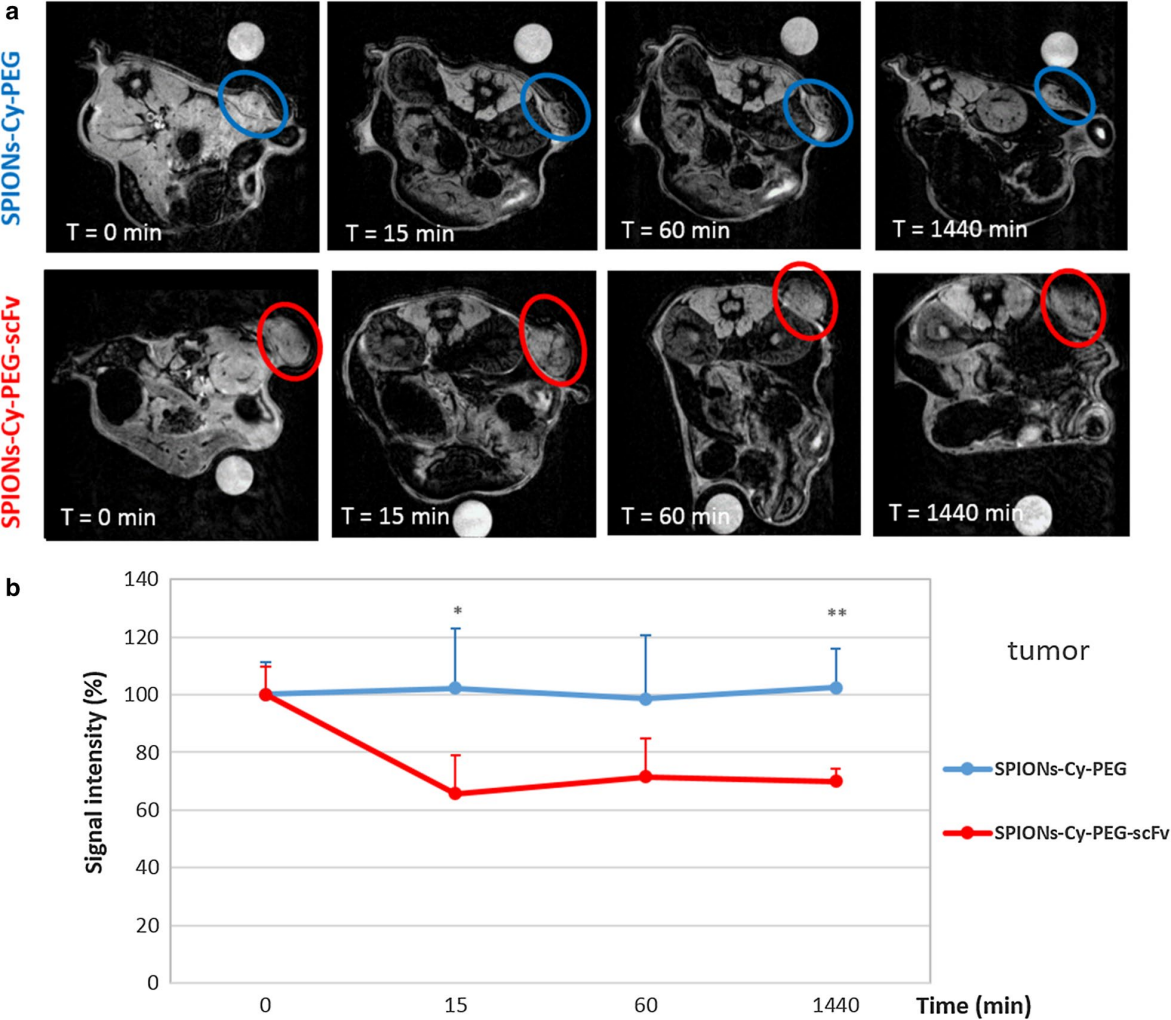
In vivo tumor targeting with SPIONs-Cy-PEG-scFv. **a** In vivo MR tumor imaging with SPIONs-Cy-PEG and SPIONs-Cy-PEG-scFv in BT-474 tumor-bearing mice (ectopic tumors were encircled for better clarity). **b** Quantification of grey level intensity in tumor after IV injection of SPIONs-Cy-PEG (blue line; n = 5) and SPIONs-Cy-PEG-scFv (red line; n = 4) (11.7 mg/kg Fe body weight) and according time. *p < 0.05, **p < 0.01 (student t-test)

**Fig. 7:**
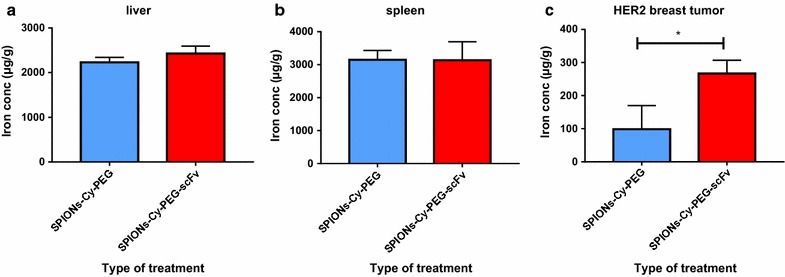
Iron concentration (in µg Fe/g organ) determined by atomic absorption spectrophotometry (AAS) in liver (**a**), spleen (**b**) and HER2-breast tumor (**c**) 24 h after the injection of SPIONs-Cy-PEG (n = 5) or SPIONs-Cy-PEG-scFv (n = 4). *p < 0.05 (Mann–Whitney test)

MR images were then acquired after the IV injection of SPIONs-Cy-PEG-scFv and SPIONs-Cy-PEG on HER2 breast tumors on mice (Fig. 6a). A quantitative evaluation was also performed by monitoring the mean grey level intensity measured on tumors (Fig. 6b) and on clearance organs (kidneys, spleen and liver) (Additional file 1: Fig. S4). As SPIONs were negative contrast agents, lower is the intensity, stronger is the accumulation in the target organ. After the injection of SPIONs-Cy-PEG, the mean grey level intensity measured on tumors stays around 100% throughout the experience showing that SPIONs-Cy-PEG weakly penetrate in HER2+ breast tumors (Fig. 6b). On the contrary, after 15 min, the grey level intensity measured for the group of mice injected with SPIONs-Cy-PEG-scFv fall down to 65 ± 13%. 15 min after the injection, the accumulation of SPION-Cy-PEG-scFv into HER2 breast tumor on mice is significant compared to mice injected with SPION-Cy-PEG (p = 0.02, student t test) showing the potential of the scFv targeting (Fig. 6b). The monitoring of grey level intensities in main clearance organs show us that SPIONs-Cy-PEG-scFv are more present in kidneys compared to SPIONs-Cy-PEG (Additional file 1: Fig. S4). As they are too large to be filtrated by kidneys [28], we make the hypothesis that SPIONs-Cy-PEG-scFv may remain longer in the kidneys irrigating bloodstream compared to SPIONs-Cy-PEG. Unfortunately, both types of nanoprobes were captured by liver and spleen. Iron quantification by AAS performed on digested organs at 24 h confirmed the results obtained by imaging (Fig. 7a, b). A high iron concentration is found in liver and spleen, whatever the nanoprobes (SPIONs-Cy-PEG or SPIONs-Cy-PEG-scFv) injected on mice, and it is not possible to make a significant difference between the two groups of mice. The amount of iron injected by mouse is equivalent to 255 µg which is very few compared to physiological iron present in liver/spleen in mice. The cellular type involved in the hepatic capture is still unknown (i.e. hepatocytes, Kupffer cells, endothelial cells, etc.) [29]. Van Beers et al. showed a significant increase in liver signal intensity on T1-weighted images until 1 h after the injection of ferumoxtran of a high dosage (150 µmol Fe/kg) whereas electron microscopy reveal the absence of NPs in hepatocytes and the appearance in Kupffer cells between 8 and 24 h after their injection [30]. It means that MRI has to be completed with optical and electron microscopies to better understand the compartmental distribution of IONP in the liver. Distribution of our SPION-Cy-PEG-scFv in liver is a subject of a new studies we currently initiate.

After 1 h, the grey level intensity measured into the HER2 breast tumor slightly increased to be around 71 ± 13% for SPIONs-Cy-PEG-scFv while it remains around 98 ± 22% for SPIONs-Cy-PEG (p = 0.08; student t-test). It means that a very few quantity of SPIONs-Cy-PEG-scFv is able to be released from the tumor. The most important is that after 24 h, the grey level in the tumor remains stable, equal to 70 ± 3% at 24 h (p = 0.0025; student t-test), for the mice injected with SPIONs-Cy-PEG-scFv. This observation shows that antibody fragments allow the retention of the nanoprobes into the tumor which is very important in the context of diagnosis. Iron quantification by AAS performed on tumors at 24 h confirmed the results obtained by imaging (Fig. 7c). Iron concentration after digestion of HER2 breast tumor was of 267 µg/g for the group of mice injected with SPIONs-Cy-PEG-scFv and about 98 µg/g for the group of mice injected with SPIONs-Cy-PEG (p = 0.0159; Mann–Whitney test). Nanoprobe functionalization with scFv anti-HER2 allows a nanoparticle retention within the tumor. In a recent study, Ding et al. investigated the feasibility of an anti-HER2 scFv-IONPs as HER2 targeted MRI contrast agents for preoperative tumor diagnosis [31]. They showed in their in vivo study, a decrease of MR signals in the tumor to 19.3% for HER2+ tumors (with N87 cells) and to 6.2% for HER2− tumors (with SUIT2 cells) 24 h after the injection of their scFv-nanoprobes. In our study, the decrease of MR signals is much better, of about 30% at 24 h after the injection of SPIONs-Cy-PEG-scFv. These results all indicate that scFv offers a high degree of specificity towards HER2 overexpressing cells/tumors and can serve as an efficient targeting ligand for magnetic imaging purposes.

## Methods

### Chemicals

Anhydrous dimethyl sulfoxyde (DMSO, 99.9%), EDTA disodium salt, *N*-hydroxysuccinimide (NHS), Dicyclohexylcarbodiimide (DCC) and Dithiothreitol (DTT) were purchased from Sigma Aldrich (Saint-Quentin-Fallavier, France). α-Maleinimidohexanoic-ω-NHS PEG, Mw 5000 Da, (NHS-PEG-Mal) was obtained from Rapp Polymere (Tübingen, Germany). Tris(2-carboxyethyl)phosphine hydrochloride (TCEP) and Coomassie Plus assay kit were purchased from Thermo Scientific (Fisher Scientific, Illkirch, France) and cyanine-5,5-NHS was obtained from Lumiprobe (Hannover, Germany). All other reagents were of analytical grade. In all the experiments, water was previously deionized (18 MΩ cm). Dialysis tubing (cellulose ester, molecular weight cut off 300 and 1000 kDa) was obtained from Spectrum Labs (France).

### Nanoparticle synthesis

The synthesis is divided into 3 steps: the fluorescence labelling of silanized SPIONs, the PEGylation of fluorescent silanized SPIONs and the functionalization of nanoparticles by scFv 4D5-Cys. For fluorescence labelling, 450 µL (6.28 µmol) of cyanine-5,5-NHS solution at 10 g/L was activated by NHS/DCC (0.126 mmol NHS and DCC) in anhydrous DMSO in the dark during 4 h, and subsequently, a suspension of 3.17 mL (16 mg or 0.287 mmol of iron) of silanized SPIONs dispersed in DMSO was added. The resulting suspension was kept under stirring in dark at room temperature during 24 h. PEGylation of fluorescent silanized SPIONs and functionalization by scFv anti-HER2 (or 4D5-Cys) is based on a protocol previously published by our group [16]. PEGylation step was performed immediately on fluorescent silanized SPIONs without an intermediate purification. Targeted fluorescent nanoprobes were named SPIONs-Cy-PEG-ScFv.

### Nanoparticle characterization

#### Size and zeta potential measurements

The mean hydrodynamic diameter and the zeta potential of nanoparticles in suspension were determined using a Malvern NanoZS (Malvern Instruments, Malvern, UK) with iron concentrations of 50 mg/L. For SPIONs-Cy-PEG-scFv (and SPIONs-Cy-PEG for comparison), hydrodynamic diameter and the zeta potential measurements were performed in PBS solvent at pH 7.4 and at 37 °C. All measurements were done at least in triplicate.

#### Iron concentration

The total iron concentration of NPs suspensions was determined by atomic absorption spectrophotometry (AAS) measurements at 248.3 nm (iCE 3000 spectrometer, Thermo Instruments, France).

#### Fluorescence emission

Emission spectra of nanoparticles-bounded cyanine-5.5 were recorded with an Edinburgh FS5 fluorescence spectrofluorometer, from 675 to 800 nm at an iron concentration of 50 mg/L. To record cyanine-5.5 emission, excitation was set to 650 nm.

#### scFv functionality

The functionality of the scFv anti-HER2, and SPIONs-Cy-PEG-scFv was checked by indirect enzyme-linked immunosorbent assays (ELISA) and by Immunofluorescence (IF). For indirect ELISA, HER2 recombinant protein (Sino Biologicals, Beijing, P. R. China.) was coated in a 96-well plates at 1 µg/mL in PBS and incubated overnight at 4 °C. The wells were then saturated with 3% BSA-PBS for 1 h at 37 °C and washed with PBS prior to incubation with PBS (negative control), SPIONs-Cy-PEG or SPIONs-Cy-PEG-scFv (from 0.003 to 100 mg L iron) during 1 h at 37 °C. Wells were then washed with PBS-Tween 20 (0.05%) and incubated with 100 µL of protein-l-peroxydase (Pierce^®^) at 1.25 µg mL for 1 h at 37 °C added to 100 µL of 3,3′,5,5′-Tétraméthylbenzidine substrate (TMB; Sigma, St Louis, USA). Enzymatic reactions were stopped with the addition of 50 µL of 1 M H_2_SO_4_ and the absorbance was measured at 450 nm using an absorbance microplate reader (Bio-Tek^®^ instruments, Inc., USA). Wells coloration correlated to the presence of scFv and the absorbance at 450 nm was then proportional to scFv content.

For immunofluorescence (IF), SK-BR3 breast cancer cells were plated at a density of 5 × 10^4^ cells/well in 24-well plates onto cover glasses for 48 h in media supplemented with serum. Cells were then washed and fixed in 4% PFA solution for 15 min at room temperature. Cover glasses surface were then saturated with a 10% FCS solution in PBS for 1 h at 37 °C. Then, the cover glasses were taken and put in contact with the samples all day night at 4 °C in a humidified chamber box. The cover glasses were washed three times with PBS and incubated for 1 h at 37 °C with protein-L-FITC (ACROBiosystems, Newark, USA) at 1.25 µg/mL for 1 h at 37 °C. Cells were finally washed with PBS and placed between slide and slip cover with 10 µL of Fluoromount G^®^ mounting medium. Observations were then made with a fluorescent inverted microscope (Olympus, IX51).

### In vitro breast cancer model

#### Cell cultures

Human breast carcinoma cells MCF-7 were obtained from the American Type Culture Collection (LGC Promochem, Molsheim, France). The cells were grown at 37 °C/5% CO_2_ in Dulbecco’s Modified Eagle Medium (DMEM) with glucose and l-glutamine containing 5% fetal bovine serum (FBS, Gibco^®^) and 1% Penicillin–Streptomycin solution (10,000 U/mL, Gibco^®^). BT-474, SK-BR-3 and MDA-MB231 cells were obtained from Cell Lines Service (CLS Eppelheim, Germany). BT-474 cells were grown at 37 °C/5% CO_2_ in DMEM:Ham’s F12 medium (1:1 mixture) supplemented with 2 mM l-glutamine, 5 µg/mL Insulin (Gibco^®^, Life Technologies) and 5% FBS. SK-BR-3 and MDA-MB231 cells were maintained in DMEM supplemented with 10% FBS, in humidified atmosphere with 5% CO_2_ at 37 °C.

#### HER2 receptor location on cells

BT-474 cells were counted and put in tubes with filter cap at a density of 2 × 10^6^ cells per tube. For the localization of HER2 receptors, cells were incubated with 100 µL of anti-HER2 microbeads (360 mg/mL of iron; Miltenyi Biotec, Paris) for 15 min at 4 °C. The cells were then concentrated by centrifugation and washed three times with PBS. Cells were then fixed in Trump’s solution consisting of phosphate buffer 0.1 M + paraformaldehyde 4% (Sigma; Steinheim, Germany) + 1% glutaraldehyde (EMS; Hatfield, PA, USA). Cells were post-fixed with 2% osmium tetroxide (EMS; Hatfield, PA, USA), dehydrated with series of increasing ethanol solutions, and embedded in Epon^®^ resin (Sigma; Steinheim, Germany). Ultrathin sections (90 nm) were stained with 2% aqueous uranyl acetate and 1% lead citrate (Merck; Darmstadt, Germany). Images were acquired using a JEOL 1011 transmission electron microscope operating at 100 kV. SK-BR3 cells were also stained with 1% of anti-human CD340 Alexa Fluor^®^ 488 (green) for 15 min at 37 °C. After that, the cells were washed and placed under microscope for examination. Cells were imaged with the SP2 confocal inverted microscope (Leica, Germany) using the water immersion 63×/1.2 NA HCX PL APO objective.

#### HER2 quantification on breast cancer cells

BT-474, SK-BR3 cells were plated at a density of 2 × 10^5^ cells/well in a 24-well plate for 48 h in culture media supplemented with FBS. MCF-7 and MDA-MB231 were plated at a density of 1.5 × 10^5^ cells/well and 1 × 10^5^ cells/well respectively in a 24-well plate for 48 h in media supplemented with FBS. After that, 10 µg/mL murine monoclonal antibody against HER2 Receptor, (clone 9G6.10; Thermo Scientific Pierce Ab) was added to cells for 30 min at 4 °C. Cells were then washed with DPBS. The primary antibody was then revealed by an Alexa Fluor^®^ 488 goat anti-mouse antibody (4 µg/mL, Molecular probes, Life technologies) for 30 min at 4 °C. Cells were washed, harvested, fixed in 2% formaldehyde and subjected to flow cytometry analysis using a MoFlo™ cell sorter (Beckman Coulter, Fort Collins, CO, USA). The Alexa Fluor^®^ 488 fluorescence was excited at 488 nm. Fluorescence emission was collected in the 530/40 nm range. About 10,000 cells were measured for each sample. Summit software (Beckman Coulter, Fort Collins, CO, USA) was used to perform flow cytometry data analysis and remove debris signals depending on morphological criteria.

#### Nanoparticle uptake by breast cancer cells

For TEM acquisitions, BT-474 cells were incubated with SPIONs-Cy-PEG and SPIONs-Cy-PEG-scFv (Fe = 180 mg/L) for 4 h in a humidified atmosphere with 5% CO_2_ at 37 °C and under constant agitation using a Stuart Tube Rotator SB2 (Bibby Scientific Ltd, UK). Then, cells were fixed in Trump’s solution and treated according to the protocol described in part 2.4.2.

For fluorescence confocal spectral imaging (CSI) measurements, BT-474 cells were plated at a density of 5 × 10^4^ cells/well onto cover glasses in 24-well plates for 48 h in media supplemented with serum. Cells were then incubated for 1 h with nanoprobes dispersed in PBS (Fe = 150 mg/L) and washed thrice with PBS. BT-474 cells were then mounted between slide and slip cover and observed by CSI in a confocal mode. Fluorescence CSI measurements were carried out using a LabRam laser scanning confocal microspectrometer (Horiba SA, Villeneuve d’Ascq, France) equipped with an automated X–Y–Z scanning stage, a low dispersion grating (300 grooves/mm) and an air-cooled CCD detector. The Cy fluorescence was excited using a 632.8 nm line of a built-in He–Ne laser. For the analysis of adherent cells, an optical section (x–y plane) situated at half-thickness of the cell was scanned with a step of 0.8 μm that provided maps containing typically 2500 spectra. Both acquisition and treatment of multispectral maps were performed with LabSpec software version 5.

For flow cytometry, BT-474, MDA-MB231 and MCF-7 were plated at a density of 2 × 10^5^, 1 × 10^5^ and 1.5 × 10^5^ cells/well respectively in a 24-well plate for 72 h in culture media supplemented with FBS. After that, SPIONs-Cy-PEG-scFv or SPIONs-Cy-PEG (150 mg/L iron) were added to cells for 15, 60, 120, 240, and 360 min at 37 °C under 5% CO_2_. Cells were then washed, harvested, washed and fixed in PBS/formaldehyde 2%. Cells were analyzed using a Gallios cytometer (Beckman Coulter). The Cy was excited at 633 nm. Kaluza software (Beckman Coulter) was used to perform flow cytometry data analysis.

### In vivo breast cancer xenograft

#### Animal model

BalB-C female nude mice (6 weeks old) were purchased from Janvier Labs (Saint Berthevin, France). BT-474 cells (10 × 10^6^ in 0.15 mL sterile PBS) were injected subcutaneously on the right flank of BalB-C nude mice under gas anesthesia. The procedures were approved by the local ethic committee (CEEA Val de Loire) and by the French Ministry of National Education, Higher Education and Research (No. 201501091617160v6). Tumor-bearing mice were separated into three distinct groups when tumor volumes were closed to 150 mm^3^.

#### In vivo tumor targeting measured by MRI

The first group of mice (n = 4) was injected with SPIONs-Cy-PEG-scFv and the second one (n = 5) with SPIONs-Cy-PEG at a final iron concentration of 11.7 mg/kg. Each preparation was injected intravenously with an external catheter connected in the caudal vein under anesthesia once the mouse was placed inside the MRI. Animals were anesthetized by inhalation of 2% isoflurane then maintained during MR experiments at 1.5% (0.5 L/min mixed in air and oxygen with 1:1 ratio). The physiological body temperature was maintained inside the magnet by warm water circulation. A pressure sensor was used to monitor the respiration cycle and obtain the respiration frequency for the Ig-FLASH (Intra-grate Fast Low Angle Shot) reconstruction.

Magnetic resonance acquisition was performed on a 9.4 Tesla Biospec 94/20 superconducting magnet (Bruker, Wissembourg, France) with a shielded gradient set (950 mT/m maximum gradient amplitude) and a transmit-receive birdcage coil with 35 mm inner diameter. A first series of axial images was performed to localize the tumor with a multislice gradient echo sequence (TE/TR = 4/105 ms, flip angle α = 20°, field of view = 4 * 4 cm, matrix size = 256 * 256, slice thickness = 1 mm) for 2 min. Then a susceptibility-weighted image (SWI) was acquired on the slice where the tumor was the biggest sequence (TE/TR = 7/300 ms, flip angle α = 30°, field of view = 3 * 3 cm, matrix size = 256 * 192, slice thickness = 1 mm, negative mask, mask weighted = 4) for 2 min. Acquired MR SWI image was transferred onto an external computer for data processing. A region of interest ROI was manually drawn as big as possible in the tumor to calculate grey levels mean. This mean was normalized with signal intensity of a ROI taken in the background.

#### Iron quantification in organs by AAS

Liver, spleen and HER2-breast tumor were excised for each mice and washed quickly with cold water to remove surface blood. Organs were first digested with a mixture of HNO_3_–HCl (1:2, V:V) for 12 h at room temperature. Then, samples were placed in a digestion system (DigiPREP MINI; SCP sciences, Courtaboeuf, France) at 100 °C during 36 h and at 120 °C during 4 h to concentrate the samples. Finally, the concentrates were diluted in water (10 mL: liver and spleen and 5 mL: tumor) and were filtered using a PES syringe filter. Iron content was then determined by AAS after an appropriate dilution.

#### Statistical analysis

All statistical data was analyzed using GraphPad Prism 7.0 software. The statistical significance was determined between groups for each time using a multiple t-test for the MRI in vivo results and using a Mann–Whitney test for iron quantification by organs (*p < 0.05, **p < 0.01).

## Additional file

**Additional file 1: Fig. S1.** Nanoprobe purification step measured by ELISA. **Fig. S2.** DLS, zeta and fluorescence characterization of nanoprobes. **Fig. S3.** HER2 quantification on breast cancer xenograft by flow cytometry. **Fig. S4.** MRI biodistribution of nanoprobes in liver, spleen and kidneys.

## Abbreviations

HER2: Human Epithelial growth Receptor 2
MRI: Magnetic Resonance Imaging
scFv: Single Chain Fragment Variable
TEM: Transmission Electronic Microscopy
SPR: Surface Plasmon Resonance
Kd: Dissociation rate constant
FACs: Fluorescence-Activated Cell sorting
CSI: Confocal Spectral Imaging
