# Supplementary material for: Targeting HER2-breast tumors with scFv-decorated bimodal nanoprobes
Source: J Nanobiotechnology. 2018 Feb 21;16:18. doi: 10.1186/s12951-018-0341-6 (PMC5820783; doi:10.1186/s12951-018-0341-6)
Supplement: Supplementary file 1 — Additional file 1: Fig. S1. Nanoprobe purification step measured by ELISA. Fig. S2. DLS, zeta and fluorescence characterization of nanoprobes. Fig. S3. HER2 quantification on breast cancer xenograft by flow cytometry. Fig. S4. MRI biodistribution of nanoprobes in liver, spleen and kidneys. [file 12951_2018_341_MOESM1_ESM.docx]

**ADDITIONAL FILE**

**
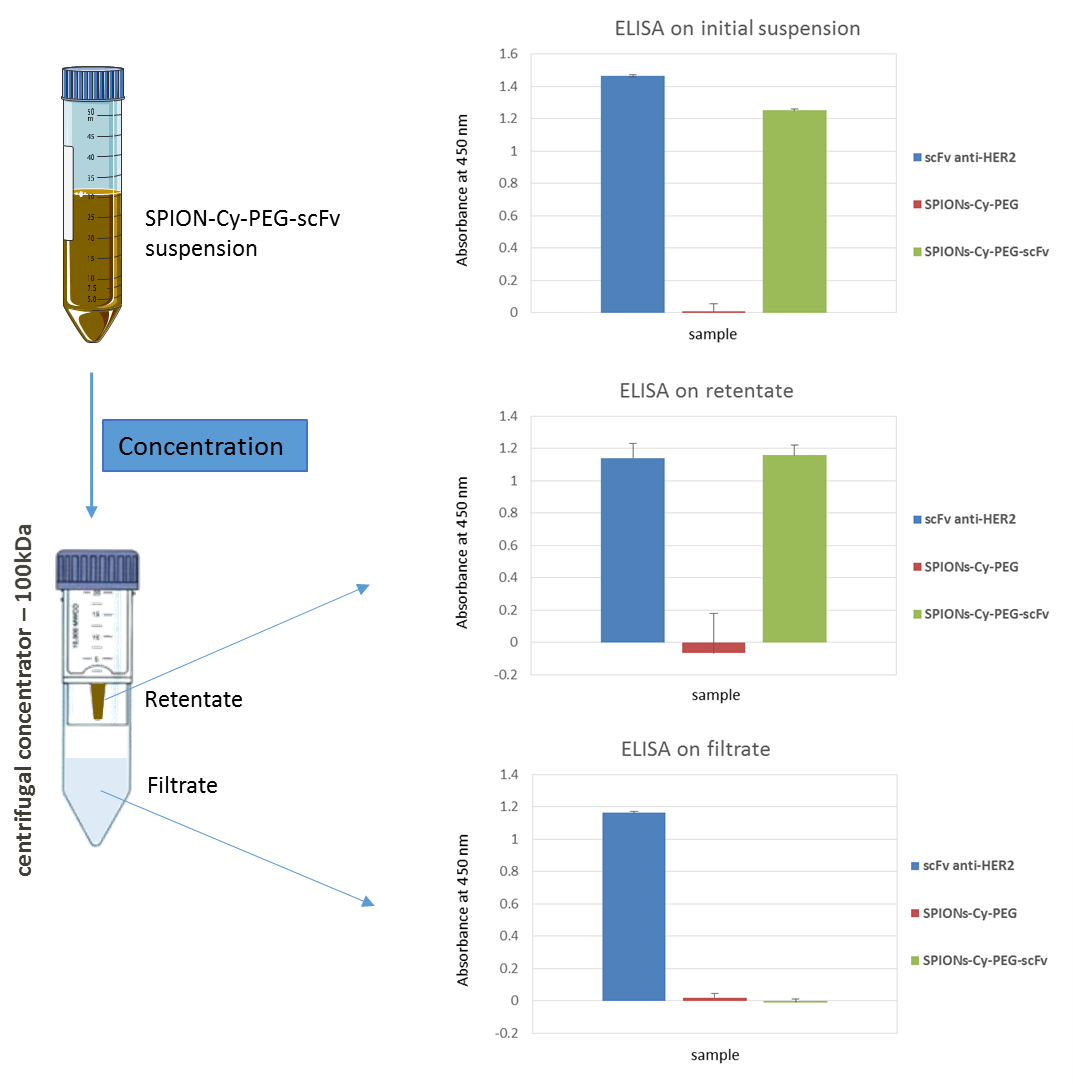
**

**Figure S1: Nanoprobe purification step measured by indirect enzyme-linked immunosorbent assays (ELISA).** Retentate and filtrate were obtained after the centrifugation of initial suspension at 3000xg for successive10 min in a centrifugal concentrator 100 kDa. ELISA measurement were made at 50 mg.L^-1^ of iron for nanoprobes and at ≈ 12 µg.mL^-1^ for anti-HER2 scFv.


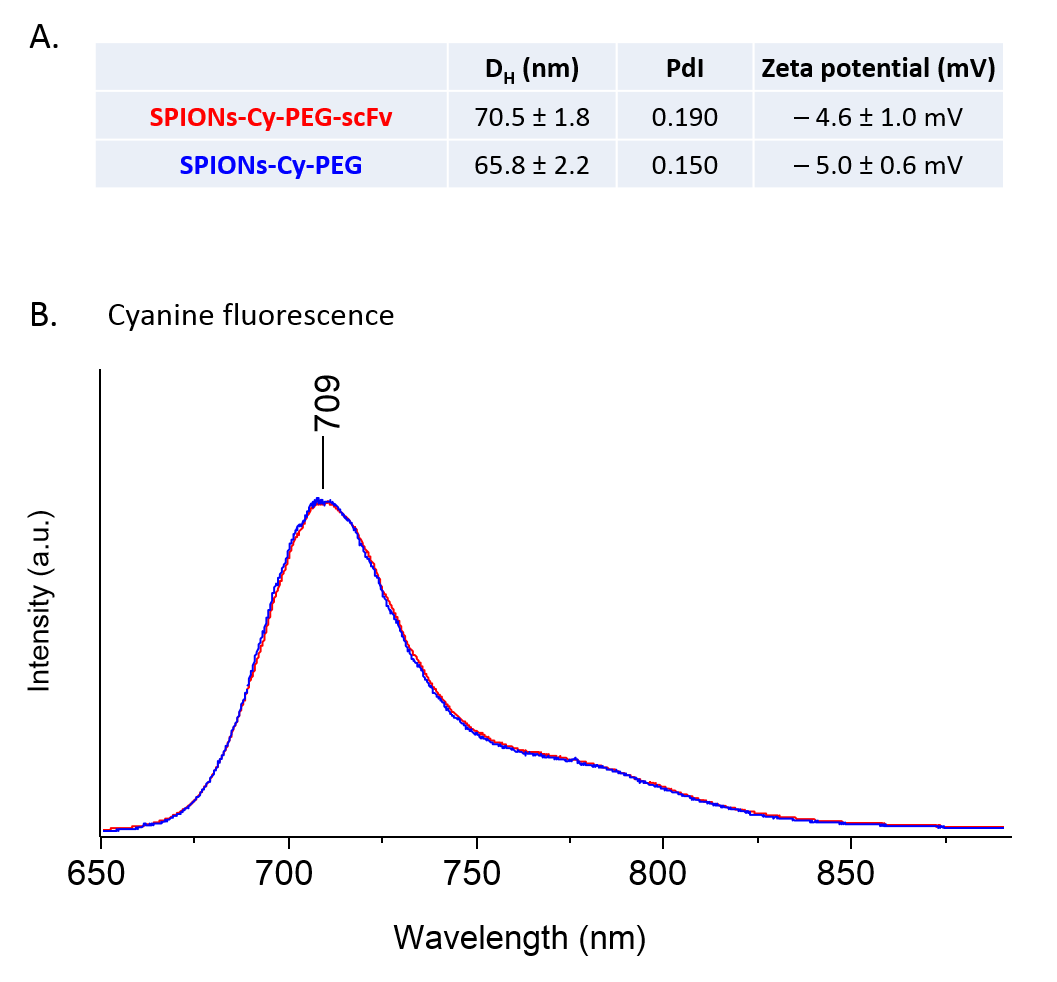


**Figure S2.** Physico-chemical characteristics of magnetic bimodal nanoprobes. A: Size measurements and zeta potential of SPIONs-Cy-PEG and SPIONs-Cy-PEG-scFv, B: Fluorescence spectra of Cyanine 5.5 covalently bound to the nanoprobes at an iron concentration of 50 mg/L. The spectra were recorded in water.


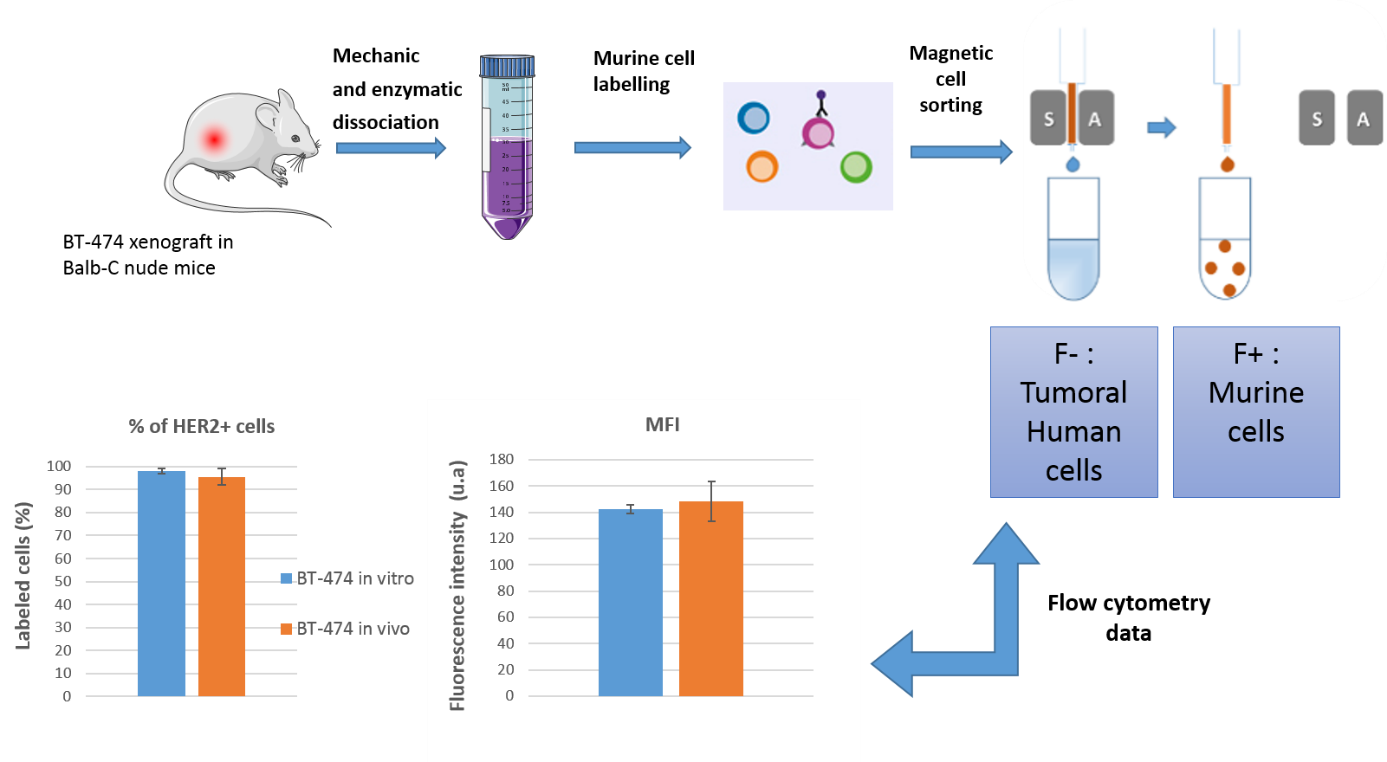


**Figure S3: HER2 quantification on breast cancer xenograft by flow cytometry.** Breast tumor xenograft were excised and put in DMEM culture media. Mechanic and enzymatic digestion were performed using a Tumor Dissociation kit^®^ (Miltenyi Biotec GmbH) with a gentleMACS dissociator® (Miltenyi Biotec GmbH). After 1 centrifugation (300 x g; 10 min), 20 µL of a mouse Cell Depletion Kit^®^ (Miltenyi Biotec GmbH) was added to the cap and put at 4 °C for 15 min. The mouse cells are magnetically labeled with a cocktail of monoclonal antibody conjugated with MACS MicroBeads. Cell suspension is then loaded onto a MACS Column and placed in the magnetic field of a MACS separator. The unlabeled human cells run through and collected for cytometry analysis as described previously in § 2.4.3.


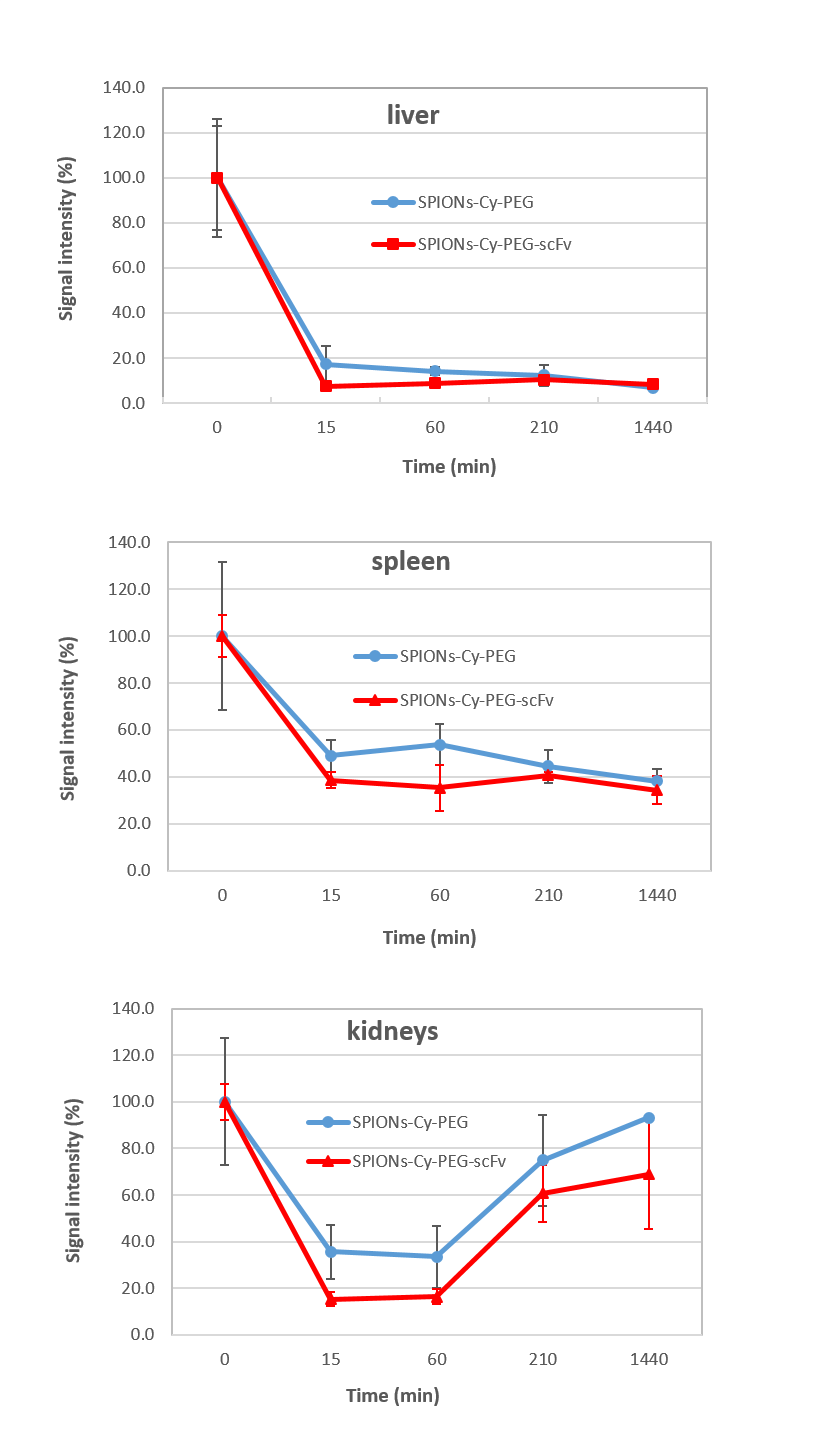


**Figure S4: Accumulation of nanoprobes in mice main clearance organs by MRI (liver, spleen and kidneys).** The accumulation was monitored by the quantification of mean grey level intensity in organs after IV injection of SPIONs-Cy-PEG (blue line) and SPIONs-Cy-PEG-scFv (red line) (11.7 mg/kg Fe body weight). Each mouse is its own control.
